# Validation study of the prognostic value of cyclin-dependent kinase (CDK)-based risk in Caucasian breast cancer patients

**DOI:** 10.1038/sj.bjc.6604870

**Published:** 2009-01-20

**Authors:** J G H van Nes, V T H B M Smit, H Putter, P J Kuppen, S J Kim, M Daito, J Ding, M Shibayama, S Numada, K Gohda, T Matsushima, H Ishihara, S Noguchi, C J H van de Velde

**Affiliations:** 1 Department of Surgery, Leiden University Medical Centre, P.O. Box 9600, Leiden 2300 RC, the Netherlands; 2 Department of Pathology, Leiden University Medical Centre, P.O. Box 9600, Leiden 2300 RC, the Netherlands; 3 Department of Statistics, Leiden University Medical Centre, P.O. Box 9600, Leiden 2300 RC, the Netherlands; 4 Department of Breast and Endocrine Surgery, Graduate School of Medicine, Osaka University, Suita 565-0871, Japan; 5 Central Research Laboratories, Sysmex Corporation, Kobe 651-2271, Japan

**Keywords:** breast cancer, cyclin-dependent kinase (CDK), distant recurrence prediction

## Abstract

In a Japanese study, cyclin-dependent kinase (CDK) based risk determined by CDK 1 and 2 activities was associated with risk of distance recurrence in early breast cancer patients. The aim of our study was to validate this risk categorization in European early breast cancer patients. We retrospectively analyzed frozen breast cancer specimens of 352 Dutch patients with histologically confirmed primary invasive early breast cancer. CDK-based risk was determined in tumour tissues by calculating a risk score (RS) according to kinases activity and protein mass concentration assay without the knowledge of outcome. Determination of CDK-based risk was feasible in 184 out of 352 (52%) tumours. Median follow-up of these patients was 15 years. In patients not receiving systemic treatment, the proportions of risk categories were 44% low, 16% intermediate, and 40% high CDK-based risk. These groups remained significant after univariate and multivariate Cox-regression analysis. Factors associated with a shorter distant recurrence-free period were positive lymph nodes, mastectomy with radiotherapy, and high CDK-based risk. There was no significant correlation with overall survival (OS). CDK-based risk is a prognostic marker of distance recurrence of patients with early breast cancer. More validation would be warranted to use of CDK-based risk into clinical practice.

Breast cancer is the most commonly occurring cancer and the leading cause of cancer death in women in the Western world (Parkin *et al*, 2005). In addition to local therapy, systemic treatment improves disease-free and overall survival (OS) in patients with early breast cancer (Early Breast Cancer Trialists' Collaborative Group, 2005). Based on traditional prognostic markers, such as age, tumour grade/size and nodal status, patients are classified into different risk groups to determine who will receive systemic treatment (Goldhirsch *et al*, 2007). However, breast cancer can recur in low-risk patients not receiving systemic treatment, resulting in a poor clinical outcome. This indicates that these conventional prognostic markers are not yet optimal for risk assessment. Although several new tumour-related biological parameters are investigated, none of these has been introduced in standard clinical practice so far (Annecke *et al*, 2008; Cardoso *et al*, 2008; Sparano and Paik, 2008).

One relevant characteristic of tumours is their aggressiveness in proliferation, which is evaluated by such biological indicators as 3H-thymidine uptake, DNA-analysis, mitotic activity index (MAI) and Ki-67 expression. These approaches are not highly useful in clinical practice, because of technical and performance instabilities. It was shown that overexpression of cyclins, which bind and activate cyclin-dependent kinases (CDKs), as well as inactivation of CDK inhibitors such as p21WAF1 and p27Kip1, which inhibit CDK activities, correlates with prognosis in a variety of malignancies (Gillett *et al*, 1994; Gramlich *et al*, 1994; Gansauge *et al*, 1997; Sutter *et al*, 1997; van Diest *et al*, 1997; Nakashima *et al*, 2000; Sjostrom *et al*, 2000; Takano *et al*, 2000; Ishihara *et al*, 2005). Therefore, direct measurement of CDK activity could provide more reliable clinical information about the prognosis than used molecular pathological parameters.

On the basis of these considerations, an assay system was developed that can directly measure the activity and expression of CDK1 and CDK2 in a routine laboratory test (SA; the kinases activity divided by the protein mass concentration) was established (Ishihara *et al*, 2005). The clinical performance of the system was first evaluated in a Japanese retrospective study in 284 early breast cancer patients with a median follow-up of almost 5 years (Kim *et al*, 2008). It was found that CDK-based risk derived from the SA of CDK1 and CDK2 was associated with risk of relapse. However, the procedure to determine this CDK-based risk is complicated and intuitive. Therefore, the data of the last Japanese study was re-evaluated to define a risk score (RS), which quantitatively indicates the risk for recurrence.

The aim of our study was to validate the prognostic value of the modified CDK-based risk recurrence model in a European patient population and to examine if CDK-based risk is correlated with established prognostic factors. In turn, these results may be used to enable better risk identification for early breast cancer patients as the basis for better risk adapted individualised adjuvant systemic treatment decisions.

## Materials and methods

### Patients

A consecutive series of patients with histologically confirmed invasive early breast cancer that received primary surgical resection in the Leiden University Medical Centre between 1985 and 1996 was used. Patients with an earlier history of cancer (other than basal cell carcinoma or cervical *in situ* carcinoma), bilateral tumours or a secondary tumour other than breast cancer, were excluded. The following data were available: age at diagnosis, histological type, TNM stage, local and systemic therapy, locoregional and distant recurrence, second primaries, and OS. All tumours were regraded by one pathologist (VS). Approval was obtained from the LUMC Medical Ethics Committee.

### Sample preparation

Tumour tissue was dissected from the surgical resection, immediately embedded in optimal cutting temperature (OCT) compound, and stored at −80°C. Ten to 20 sections of 100 μm thickness were cut from the embedded tissue with a cryostat and subjected to CDK analysis as described below. To analyze the influence of OCT contamination to the assay system, the OCT content at the surface of the cryosection was recorded as a percentage.

### Determination of CDK-specific activities

The system to measure the CDK SA is named C2P® (Sysmex, Kobe, Japan). In brief, lysate of frozen material was applied to a well of a dot-blot device. Expression of CDKs was detected quantitatively by sequential reactions with primary anti-CDK antibodies, biotinylated anti-rabbit antibodies, and fluorescein-labelled streptavidin. To measure kinases activity, each CDK molecule was immunoprecipitated from the tissue lysate. The thiophosphate of ATP-*γ*S was transferred to the protein substrate during the on-bead kinases reaction. The introduced thiophosphate was labelled further with 5-iodoacetamidofluorescein and blotted onto a polyvinylidene fluoride membrane. The kinases activity was determined by measuring the fluorescence intensity of the blot.

CDK SA was calculated as kinases activity (mAU per *μ*l lysate) divided by its corresponding mass concentration (EU per *μ*l lysate). Both of AU (CDK activity unit) and EU (CDK expression unit) were defined as the equivalent expression and activity of 1 ng of recombinant active CDK molecule, respectively.

### Risk score

In the previous Japanese study, the distribution of CDK1SA and CDK2SA was moderately related (*r*=0.501, log (CDK2SA)=0.533log (CDK1SA)+1.225), and the aberration from this relationship correlated with the rate of recurrence (Kim *et al*, 2008). Besides, recurrences were frequently observed in patients with a tumour with higher CDK1SA. The extent of the aberration was quantified as the ratio of CDK2SA relative to CDK1SA (CDK2SA/CDK1SA). The rate of recurrence monotonically increased with increasing ratio of CDK2SA/CDK1SA or CDK1SA. These plots approximated logistic curves (Equations (1) and (2)) and the *RS* was defined by combining these relational equations. Setting the cutoff value with the cases of the Japanese study; 40% of the patients in the high *RS* group, showed a significantly lower recurrence-free survival rate in 5 years after surgery (84.0%; 9 out of 58) compared with 40% of the low *RS* group (96.5%; 2 out of 58) (*P*=0.009). Less than 20% of the patients were regarded as an intermediate *RS* group.

The risk for recurrence was quantified as an *RS*. 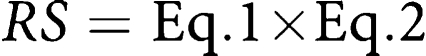


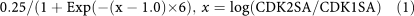


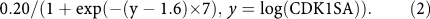


### Exclusion from statistical analysis

Severe blood contamination into the tissue lysate impairs the accuracy of the expression analysis. To avoid this problem, the extent of contamination is routinely visually quantified by comparing the redness of the lysate with a standard colour bar, which ranges from dark to faint and is graded 1–10; tissue with grade 1–3 are excluded from analysis. Another sample was excluded due to assay failure. Cellularity of the tissue was judged in the C2P system by the expression of CDKs because the molecule is expressed ubiquitously and continuously during the cell cycle. All samples whose CDK1 or CDK2 expression was below the detection limit of the system (3.2 U *μ*l of lysate for CDK1 and 0.08 U *μ*l of lysate for CDK2) were judged to contain an insufficient number of cells for the system and were excluded from the analysis.

### Statistical analysis

Statistical analyses were performed using the statistical package SPSS for Windows 15.0. (SPSS Inc., Chicago, IL, USA). Descriptive data are given as mean (s.d.) or median (range). The relationship between CDK-based risk groups and established prognostic factors were investigated using Pearson's *χ*^2^ test. All testing was two-tailed with 0.05 as level of significance (Altman *et al*, 2000).

Distant Recurrence-Free Period (DRFP) was defined as the time from surgery up to the first date of distant recurrence. Overall Survival was defined from the date of surgery up to the date of death due to any cause. To examine if CDK-based risk correlates with DRFP and OS, univariate Cox analysis was performed. Multivariate analyses were performed using the Cox-proportional hazards model entering CDK with other significant variables (defined as those with *P*<0.1 on univariate analysis). Distance Recurrence Free Period rates are reported as cumulative incidence functions, after accounting for death as competing risk (Putter *et al*, 2007).

### Role of funding source

This retrospective study was sponsored by an unrestricted educational grant of Sysmex (Kobe, Japan).

## Results

### Patients

A total of 803 patients with early breast cancer were treated with primary surgery in our centre during the study period. Frozen material was available from 352 out of 803 (44%) patients. Median follow-up of patients alive at last follow up was 15 years (range, 6–21). Clinicopathological and treatment characteristics are shown in Table 1. There were minor differences between patients in which CDK was feasible and in which it was not possible. CDK determination was feasible in those patients who had larger tumours (70 *vs* 60%), had slightly more node-positive disease (53 *vs* 47%) and were less treated with breast conserving surgery (24 *vs* 35%). In general, there were no large differences in survival characteristics.

### CDK1- and 2-specific activities in tumour tissue

Determination of CDK-based risk by RS was successful in 52% of patients (184 out of 352). In 48% of cases it was not possible due to extreme blood contamination (*n*=33), OCT contamination (*n*=45), assay failure (*n*=1) or low cellularity (*n*=79). According to CDK-based risk, 41% (*n*=76) were classified as low, 13% (*n*=23) as intermediate, and 46% (*n*=85) as high CDK-based risk.

### CDK-based risk and clinicopathological parameters

Correlation between established clinicopathological variables and CDK-based risk are shown in Table 2. There was a significant association between CDK-based risk and age, nodal status, and grade. High CDK-based risk was increasingly evident in younger patients, node-positive disease and grade-III tumours. There was also an association between histological type and CDK-based risk; however, most tumours were ductal carcinomas. No significant association was found between CDK-based risk and tumour size, hormonal receptors, Ki-67 expression, HER2 expression, and vascular invasion.

### CDK-based risk and survival

**Figure 1:**
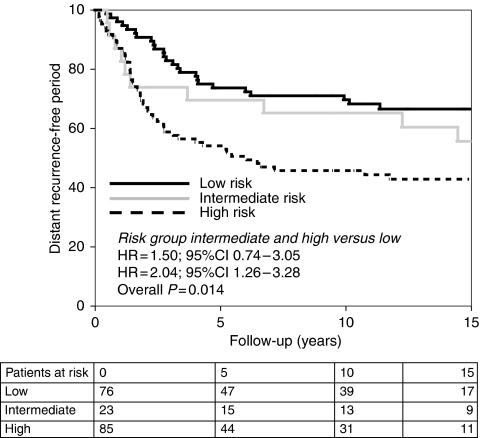
Distant recurrence-free period according to CDK-based risk. Patients with a low CDK-based risk have a longer distant recurrence-free period than patients with a high CDK-based risk.

Patients with tumours classified as low or intermediate CDK-based risk showed higher DRFP rates than patients with tumours with high CDK-based risk (intermediate and high-risk group *vs* low-risk group; hazard ratio (HR) 1.50; 95% confidence intervals (CI) 0.74–3.05 and HR=2.04; 95% CI 1.26–3.28, respectively, overall *P*-value=0.014) (Figure 1). If we compare the low *vs* high CDK-based risk group concerning DRFP at 5, 10 and 15 years, 95% CIs are 0.04–0.34, 0.07–0.38 and 0.06–0.38, respectively. Patients with a low CDK-based risk have a better OS than patients with a high CDK-based risk, although this difference is not statistically significant (intermediate and high-risk group *vs* low-risk group; HR=0.94; 95% CI 0.49–1.79 and HR=1.37; 95% CI 0.92–2.05, respectively, overall *P*-value=0.216).

All variables considered important for DRFP were analysed in Cox analysis (Table 3). In multivariate Cox-regression analysis, positive nodal status, mastectomy with radiotherapy, no systemic treatment, and high CDK-based risk were predictive for a decreased DRFP.

### Prognostic value of CDK-based risk

**Figure 2:**
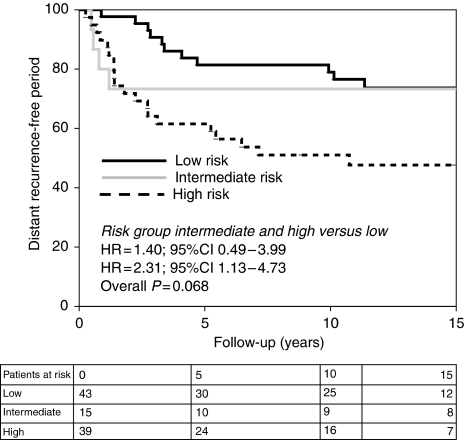
Distant recurrence-free period according to CDK-based risk in patients treated with local therapy only. Patients with a low CDK-based risk have a longer distant recurrence-free period than patients with a high CDK-based risk.

To examine the prognostic value of CDK-based risk, we excluded all patients who received systemic treatment. This patient population could be categorised by CDK-based risk as: low 44% (*n*=43 out of 97), intermediate 16% (*n*=15 out of 97), and high 40% (*n*=39 out of 97). Patients with tumours classified as low or intermediate CDK-based risk showed higher DRFP rates than patients with tumours with high CDK-based risk (intermediate and high-risk group *vs* low-risk group; HR 1.40; 95% CI 0.49–3.99 and HR=2.31; 95% CI 1.13–4.73, respectively, overall *P*-value=0.068) (Figure 2). If we compare the low *vs* high CDK-based risk group, differences in DRFP at 5, 10, and 15 years are 19, 28 and 26%, respectively. Accompanying 95% CIs for these differences are 0.01–0.39, 0.06–0.48, and 0.01–0.47, respectively (Figure 2). There was no statistical difference between these groups concerning OS (intermediate and high-risk group *vs* low-risk group; HR=1.06; 95% CI 0.47–2.37 and HR=1.44; 95% CI 0.81–2.54, respectively, overall *P*-value=0.432).

Univariate and multivariate Cox-regression analysis showed that CDK-based risk groups remained statistically significant (Table 4). Factors associated with a shorter DRFP were positive lymph nodes, mastectomy with radiotherapy, and high CDK-based risk.

## Discussion

In our European patient population with early breast cancer, the CDK-based risk was validated. Multivariate analysis showed that CDK-based risk was an independent significant prognostic factor for DRFP in all patients and in patients treated with local therapy only. CDK-based risk is a tangible prognostic marker for DRFP.

Currently, risk-assessments with various prognostic and predictive markers are used for indication of systemic treatment, like tumour grade and nodal status for general systemic treatment choice, hormonal receptors for hormonal treatment, and HER2 expression for immunotherapy. However, these assessments are insufficient for optimal therapeutic decision, especially when applied to node-negative early breast cancer patients. Only few of these patients are considered at such a low-risk of relapse that systemic therapy can be avoided. At the same time, not all patients at high-risk experience a recurrence. Therefore, there is demand for more accurate prognostic markers for a more tailored definition of an individual patient's risk of disease recurrence and to identify indications for the best therapy.

In 2007, the American Society of Clinical Oncology Committee recommended the following markers in clinical practice in patients with early breast cancer: ER, PgR, HER2, urokinases plasminogen activator (uPA), plasminogen activator inhibitor-1 (PAI-1), and certain genes detected with multiparameter gene expression assays (Harris *et al*, 2007). ER, PgR, and HER2 are widely used and should be determined in every patient with early breast cancer. uPA and PAI-1 are key factors in efficient focal proteolysis, adhesion, and migration of tumour cells (Andreasen *et al*, 1997; Schmitt *et al*, 1997; Bouchet *et al*, 1999; Duffy, 2002; Harbeck *et al*, 2002). Currently, the prognostic value of uPA and PAI-1 are being examined in the prospective Node-Negative Breast Cancer III (NNBC 3)-Europe Trial (Annecke *et al*, 2008). As another prognostic tool, the value of microarray-based prognostics and feasibility of its clinical application into clinical practice is in the process of evaluation by two major trials. The first prospective trial is the European Microarray in Node-Negative Disease May Avoid Chemotherapy Trial evaluating MammaPrint (Agendia, Amsterdam, the Netherlands), a 70-gene expression profile, in node-negative early breast cancer patients (van de Vijver *et al*, 2002; Cardoso *et al*, 2008). Its American counterpart, the Trial Assigning Individualized Options for Treatment, is aimed at validating Oncotype DX (Genomic Health, Redwood City, CA, USA), a 21-gene assay, likewise in node-negative patients (Palli *et al*, 1999; Sparano and Paik, 2008). The Oncotype DX profile can be determined using paraffin-embedded breast tissue; the MammaPrint profile makes use of fresh frozen material. Both profiles must be analysed centrally; no ready-to-use kit is available to determine the profile in local hospitals.

In the ideal clinical trial setting, the above-mentioned prognostic factors, including CDK-based risk, should be determined in the same tumour sample to determine the best marker combination for optimal treatment decisions. Unfortunately it is not likely that such a large, long-lasting and costly trial will be actualised.

From our results, it was shown that validation of CDK-based risk was feasible for European patients even though the RS was determined in Japanese patients. Despite the difference between the cohorts, it may be concluded that CDK-based risk is a new prognostic factor. However, before this CDK-based risk can be used in daily clinical practice, several aspects have to be considered. First issue is the high proportion of exclusion cases in this study; in almost 50% of tumours it was not feasible to determine CDK-based risk. This was related to weaknesses of the assay system, because the accuracy of the expression analysis is influenced by blood and OCT contamination. However, we believe these issues are not significant problems anymore. A washing step before tissue lysis markedly improved the efficacy of CDK expression analysis for cases with ⩾20% OCT contamination (data not shown). The crucial issue of the exclusion is low cellularity measured by CDK expression. In our study, 22% (79 out of 352) were excluded because of low cellularity. The Japanese retrospective study, which used snap-frozen tissues, found a lower rate of low cellularity (11%, unpublished data), and this value was judged as a clinically practical value. One challenge is to carefully determine the sufficient amount of a cryosection to apply CDK-based risk on OCT-embedded samples. Secondly, to enable general use for broad application, a feasible ready-to-use kit should be devised including the cell cycle profile system.

In the association analysis of CDK-based risk to clinicopathologic factors, we found significant associations between the CDK-based risk and age, nodal status, and grade. Unexpectedly, a statistically significant association was not observed between CDK-based risk and Ki67 expression, known as a proliferation marker of tumour tissues. This discrepancy may be because of the technical issue of Ki67 immunohistochemistry procedure; the lack of an international standardisation method for antigen retrieval, staining procedures and scoring methods. To further address the significance of CDK-based analysis, cell biological interest for rate of cell proliferation, cell cycle distribution (G1/S/G2M-phase), check point regulation, and CDK-mediated cell death should be examined both *in vitro* and *in vivo*.

In conclusion, our results showed that CDK-based risk is prognostic for DRFP in patients with early breast cancer, also after correction with other prognostic factors. Therefore, further studies are justified to develop this as a marker for more tailored treatment of early breast cancer patients.

## Figures and Tables

**Table 1:** Patients/tumour/treatment characteristics.

	**Patients with fresh frozen tumour tissue available**		**Same as previous column, in which CDK determination was feasible**		**Same as previous column, but patients received no systemic treatment**	
	***N***	**%**	***N***	**%**	***N***	**%**
*Age (years)*						
<40	28	8	16	9	7	7
40–50	90	26	45	25	21	22
51–60	74	21	39	21	19	20
>60	160	46	84	46	50	52
						
*Tumour size*						
< 20 mm	142	40	55	30	38	39
⩾20 mm	210	60	129	70	59	61
						
*Tumour grade*						
I	57	17	22	12	13	14
II	163	48	86	48	49	53
III	120	35	71	40	31	33
						
*Histological type*						
Ductal	318	94	169	94	88	95
Lobular	20	6	9	5	4	4
Other	2	1	1	1	1	1
						
*Nodal stage*						
Negative	186	53	87	47	76	78
Positive	166	47	97	53	21	22
						
*Oestrogen receptor*						
Positive	87	39	52	43	24	36
Negative	137	61	70	57	42	64
						
*Progesterone receptor*						
Positive	104	46	65	52	33	49
Negative	123	54	60	48	34	51
						
*Ki-67 expression*						
<5	143	63	85	69	47	71
⩾5	84	37	39	32	19	29
						
*HER2*						
0+/1+	198	88	101	84	53	82
2+/3+	28	12	20	17	12	19
						
*Vascular invasion*						
Negative	287	84	149	83	81	86
Positive	54	16	31	17	13	14
						
*Local treatment*						
MST without radiotherapy	151	43	86	47	58	60
MST with radiotherapy	78	22	53	29	14	14
BCS without radiotherapy	5	1	2	1	0	0
BCS with radiotherapy	118	34	43	23	25	26
						
*Systemic treatment*						
Chemotherapy alone	65	19	42	23	0	0
Hormonal therapy alone	66	19	36	20	0	0
Both	15	4	9	5	0	0
None	206	59	97	53	97	100
						
*Survival*						
Locoregional recurrence	44	13	21	11	10	10
Distant recurrence	146	42	85	46	37	38
Death	197	56	110	60	56	58
						
Total	352	100	184	100	97	100

BCS=breast conserving surgery; MST=mastectomy.

The numbers and percentage are calculated on available data. Missing data are not shown.

First of all patients treated in the Leiden University Medical Centre between 1985 and 1996 with available fresh frozen tumour tissue, second of patients in which cyclin dependent kinase (CDK) determination was feasible, third of patients in which CDK-determination was feasible and who were only treated with local therapy.

**Table 2:** Association between cyclin dependent kinase (CDK)-based risk groups and well established prognostic factors

	**CDK-based risk**		**CDK-based risk**		**CDK-based risk**		
	**Low**		**Intermediate**		**High**		
	***N***	**%**	***N***	**%**	***N***	**%**	***P*-value**
*Age (years)*							0.022
<40	3	19	1	6	12	75	
40–50	13	29	4	9	28	62	
50–60	20	51	6	15	13	33	
>60	40	48	12	14	32	38	
							
*Tumour size*							0.261
<20 mm	23	42	10	18	22	40	
⩾20 mm	53	41	13	10	63	49	
							
*Nodal stage*							0.01
Negative	43	49	14	16	30	35	
Positive	33	34	9	9	55	57	
							
*Tumour grade*							0.01
I	8	36	4	18	10	46	
II	44	51	14	16	28	33	
III	23	33	5	7	43	61	
							
*Histological type*							0.046
Ductal	69	41	20	12	80	47	
Other	6	60	3	30	1	10	
							
*Oestrogen receptor*							0.123
Negative	17	33	6	12	29	56	
Positive	32	46	12	17	26	37	
							
*Progesterone receptor*							0.183
Negative	21	32	11	17	33	51	
Positive	29	48	7	12	24	40	
							
*Ki-67 expression*							0.387
<5	37	44	12	14	36	42	
⩾5	12	31	6	15	21	54	
							
*HER2*							0.444
Negative	40	40	16	16	45	45	
Positive	9	45	1	5	10	50	
							
*Vascular invasion*							0.84
Negative	62	42	20	13	67	45	
Positive	13	42	3	10	15	48	

High CDK-based risk is higher in younger patients, node-positive disease and grade III tumours.

**Table 3:** Univariate and multivariate analysis of distant recurrence-free period (DRFP). Independent factors for a shorter DRFP are positive nodal status, mastectomy with radiotherapy, no systemic treatment and intermediate/high CDK-based risk

		**Univariate analysis**			**Multivariate analysis**		
**Characteristics**	***N***	**HR**	**95% CI**	***P*-value**	**HR**	**95% CI**	***P*-value**
*Age (years)*							
<50	61	1.00		0.720			
⩾50	123	0.92	0.59–1.44				
							
*Tumour size*							
<20 mm	55	1.00		0.013	1.00		0.660
⩾20 mm	129	1.96	1.15–3.34		1.21	0.52–2.84	
							
*Nodal status*							
Negative	87	1.00		0.000	1.00		0.004
Positive	97	3.08	1.92–4.95		2.86	1.40–5.87	
							
*Grade*							
I/II	108	1.00		0.231			
III	71	1.30	0.85–2.01				
							
*Histological type*							
Ductal	169	1.00		0.967			
Other	10	1.02	0.41–2.52				
							
*Local therapy*							
MST−RT	86	1.00		0.000	1.00		0.003
MST+RT	53	2.35	1.46–3.78		2.67	1.36–5.25	
BCT	45	0.80	0.34–1.46		0.73	0.31–1.73	
							
*Oestrogen receptor*							
Negative	52	1.00		0.222			
Positive	70	1.40	0.81–2.42				
							
*Progesterone receptor*							
Negative	65	1.00		0.512			
Positive	60	0.84	0.51–1.40				
							
*Ki-67 expression*							
<5	85	1.00		0.100	1.00		0.618
⩾5	39	1.56	0.92–2.64		1.15	0.66–2.01	
							
*HER2 expression*							
Negative	101	1.00		0.873			
Positive	20	0.94	0.46–1.93				
							
*Lymphagio invasion*							
Negative	149	1.00		0.039	1.00		0.699
Positive	31	1.70	1.03–2.82		1.15	0.56–2.38	
							
*Systemic therapy*							
No	97	1.00		0.020	1.00		0.011
Yes	87	1.66	1.08–2.56		0.40	0.20–0.81	
							
*CDK-based risk*							
Low	76	1.00		0.014	1.00		0.023
Intermediate	23	1.50	0.74–3.05		1.89	0.82–4.39	
High	85	2.04	1.26–3.28		2.36	1.27–4.37	

BCT=breast conserving therapy; MST=mastectomy; RT=radiotherapy.

**Table 4:** Univariate and multivariate analysis of distant recurrence-free period (DRFP) for patients receiving only local treatment to examine the real prognostic value of CDK-based risk

		**Univariate analysis**			**Multivariate analysis**		
**Characteristics**	***N***	**HR**	**95% CI**	***P*-value**	**HR**	**95% CI**	***P*-value**
*Age (years)*							
<50	28	1.00		0.237			
⩾50	69	1.58	0.74–3039				
							
*Tumour size*							
<20 mm	38	1.00		0.065	1.00		0.582
⩾20 mm	59	1.98	0.96–4.10		1.26	0.56–2.83	
							
*Nodal status*							
Negative	76	1.00		0.000	1.00		0.004
Positive	21	3.50	1.81–6.77		2.70	1.37–5.34	
							
*Tumour grade*							
I/II	62	1.00		0.467			
III	31	1.28	0.66–2.51				
							
*Histological type*							
Ductal	88	1.00		0.352			
Other	5	0.39	0.05–2.85				
							
*Local therapy*							
MST-RT	58	1.00		0.000	1.00		0.000
MST+RT	14	4.98	2.36–10.51		4.71	2.06–10.79	
BCT	25	0.53	0.21–1.36		0.46	0.16–1.29	
							
*Oestrogen receptor*							
Negative	24	1.00		0.788			
Positive	42	1.12	0.50–2.48				
							
*Progesterone receptor*							
Negative	33	1.00		0.934			
Positive	34	0.97	0.47–2.01				
							
*Ki-67 expression*							
<5	47	1.00		0.165			
⩾5	19	1.72	0.80–3.70				
							
*HER2 expression*							
Negative	53	1.00		0.166			
Positive	12	1.84	0.78–4.37				
							
*Vascular invasion*							
Negative	81	1.00		0.049	1.00		0.057
Positive	13	2.20	1.00–4.84		2.32	0.97–5.51	
							
*CDK-based risk*							
Low	43	1.00		0.068	1.00		0.018
Intermediate	15	1.40	0.49–3.99		2.33	0.76–7.15	
High	39	2.31	1.13–4.73		2.99	1.40–6.39	

BCT=breast conserving therapy; MST=mastectomy; RT=radiotherapy.

Independent factors for a shorter DRFP are positive nodal status, mastectomy with radiotherapy and intermediate/high CDK-based risk.
